# Management of dental patients receiving antiplatelet therapy or chronic oral anticoagulation: A review of the latest evidence

**DOI:** 10.1080/13814788.2017.1350645

**Published:** 2017-07-26

**Authors:** Csaba András Dézsi, Balázs Bence Dézsi, András Döme Dézsi

**Affiliations:** ^a^ Department of Cardiology, Petz Aladár County Teaching HospitalGyőrHungary; ^b^ Private Practice, SolyDent Dentist’s OfficeGyőrHungary; ^c^ Department of Cardiology, State Hospital for CardiologyBalatonfüredHungary

**Keywords:** Antiplatelet therapy, vitamin K antagonists, direct oral anticoagulant, dental interventions, periprocedural management

## Abstract

The perioperative management of patients treated with antithrombotic medications who undergo surgical procedures represents a common clinical problem. Dental interventions are usually associated with a low risk of bleeding; however, the dental implications of new antithrombotic agents are not yet fully understood. The present review is based on the latest evidence and recommendations published on the periprocedural management of dental patients treated with single or dual antiplatelet therapy, vitamin K antagonists, or direct oral anticoagulants for a variety of indications.

Key messages

- Most dental interventions can be safely performed without the alteration of antiplatelet therapy or anticoagulant therapy in patients taking direct oral anticoagulants or vitamin K antagonists.
- Before high bleeding risk procedures, missing one dose of direct oral anticoagulants on the morning of the intervention may be recommended.

## Introduction

Most practical recommendations consider dental procedures as minor interventions associated with a low risk of bleeding and self-limited blood loss that can be managed with local haemostatic agents [1–3]. However, certain interventions, such as dental reconstruction surgery, may require the temporary discontinuation of antithrombotic therapy. Therefore, it may not be appropriate to handle dental procedures as a homogeneous group when it comes to assessing the risk of bleeding. The Scottish Dental Clinical Effectiveness Programme (SDCEP) guidance provides a comprehensive classification of dental interventions based on the associated bleeding risks (Table 1) [2].

**Table 1.** A comprehensive classification of dental interventions based on the associated bleeding risks as recommended by the Scottish Dental Clinical Effectiveness Programme (SDCEP) [2].

	Dental procedures that are likely to cause bleeding	
Dental procedures that are unlikely to cause bleeding	Low bleeding risk procedures	High bleeding risk procedures
•Local anaesthesia by infiltration, intraligamentary or mental nerve block •Local anaesthesia by inferior dental block or other regional nerve blocks •Basic periodontal examination (BPE) •Supragingival removal of plaque, calculus, and stain •Direct or indirect restorations with supragingival margins •Endodontics (orthograde) •Impressions and other prosthetic procedures •Fitting and adjustment oforthodontic appliances	•Simple extractions (1–3, with restricted wound size) •Incision and drainage of intraoral swellings •Detailed six-point full periodontal examination •Root surface instrumentation (RSI) •Direct or indirect restorations with subgingival margins	•Complex extractions, adjacent extractions that will cause a large wound, or more than three extractions at once •Flap raising procedures ^ Elective surgical extractions ^ Periodontal surgery ^ Preprosthetic surgery ^ Periradicular surgery ^ Crown lengthening ^ Dental implant surgery •Gingival recontouring •Biopsies

Due to the increasing life expectancy and the ageing of the population, the periprocedural management of patients receiving oral anticoagulant or antiplatelet therapy for the primary or secondary prevention of cardiovascular disease is an increasingly common clinical problem [4,5]. The management of these patients represents a challenge for physicians as they should carefully balance the risk of bleeding with the risk of thromboembolic complications resulting from the temporary interruption of antithrombotic therapy. Previous studies have demonstrated that in the case of dental procedures, the risk of thrombotic events due to altering or discontinuing antithrombotic therapy far outweighs the low risk of potential perioperative bleeding complications among patients treated with single or dual antiplatelet therapy or vitamin K antagonists [6–11].

However, less is published on the management of dental patients receiving direct oral anticoagulants (DOAC) and novel oral antiplatelet (NOAC) agents, the dental implications of which have only been investigated since 2012 [12]. The management approaches followed by dental practitioners in these patients show significant variations and inconsistencies, which reflects the lack of large-scale studies and evidence-based recommendations in this setting [13,14]. Furthermore, a recent survey demonstrated the lack of current evidence and clear guidance to oral surgeons and general dental practitioners on the management of patients taking dual antiplatelet therapy (DAPT) requiring dentoalveolar surgical procedures [15]. Another recent survey has revealed that although dentists are aware of the periprocedural management of traditional anticoagulants and antiplatelet agents, there was a significant lack of knowledge about the new agents. Moreover, the results suggest that most dentists overestimate the risk of bleeding, which underlines the importance of dental education programmes and further training in this setting [16].

Therefore, the primary aim of this article is to provide a summary of the latest relevant evidence on the periprocedural antithrombotic management of patients undergoing dental procedures, intending to help dentists’ and general practitioners’ decision-making in this setting. For this purpose, a comprehensive search of the literature was performed through PubMed using ‘dabigatran,’ ‘rivaroxaban,’ ‘apixaban,’ ‘edoxaban,’ ‘warfarin,’ ‘antiplatelet,’ ‘dental,’ ‘oral,’ ‘surgery’ as search terms. Studies that provided general and specific information on the management of oral anticoagulants and antiplatelet agents in the perioperative setting and a dental context were identified and selected.

## Dental patients receiving single or dual antiplatelet therapy (SAPT or DAPT)

A range of oral antiplatelet drugs is available for managing conditions associated with the cardio- and cerebrovascular systems, which can be used both individually (SAPT) and in combination as dual antiplatelet therapy (DAPT). Dual antithrombotic regimens consisting of low-dose acetylsalicylic acid and P2Y_12_ inhibitors, such as clopidogrel or the new agents ticagrelor and prasugrel being recommended as first-line, are the mainstay to reduce the risk of recurrent ischaemic events during the first year after acute coronary syndrome (ACS) [17,18]. Furthermore, DAPT is widely used following percutaneous coronary intervention (PCI) with stenting, in patients with symptomatic peripheral vascular disease undergoing percutaneous lower extremity revascularization as well as for the prevention of recurrent stroke [19].

Previous studies suggest that the risks of thrombotic events due to altering or discontinuing the use of single or dual antiplatelet therapy far outweigh the low risk of postoperative oral bleeding complications resulting from low bleeding risk dental procedures and those that are unlikely to cause bleeding [6,7]. Therefore, minor interventions such as simple dental extractions with limited wound size may be safely performed in patients receiving single or dual antiplatelet therapy (Table 2) [20]. Furthermore, a systematic review of antiplatelet therapy and dental procedures found no clinically significant increased risk of postoperative bleeding complications from invasive dental procedures (tooth extractions [single and/or multiple, including third molar extractions], alveoloplasty, apicoectomy, implant placement, torus removal, excisional biopsies, flap surgery, periodontal surgery, and deep scaling and root planning) in patients on either single or dual antiplatelet therapy [21]. Accordingly, the alteration or discontinuation of single or dual antiplatelet therapy consisting of acetylsalicylic acid and clopidogrel is not recommended for any dental procedures [22,23]. For high bleeding risk procedures, the use of local haemostatic measures is recommended. However, it has to be noted that there is limited evidence on the intraoperative and postoperative pharmacodynamics of prasugrel and ticagrelor in dental and oral surgery [24]. Further research is required to determine whether discontinuation is required before minor dental interventions as these novel agents are increasingly incorporated into everyday clinical practice.

**Table 2.** Periprocedural recommendations in case of dental procedure.

	Periprocedural recommendations		
Presumed bleeding risk of procedure	SAPT/DAPT with ASA ± clopidogrel	VKA	DOACs
Unlikely to cause bleeding	Perform dental procedure without interruption	Perform dental procedure without interruption. if INR is ≤3.5 24 hours before the intervention.	Continue therapeutic anticoagulation, perform dental procedure at trough concentrations
Low bleeding risk dental procedures	Perform dental procedure without interruption	Perform dental procedure without interruption if INR is ≤3.5 24 h before the intervention. Delay if INR >3.5 and adjust VKA dose until INR ≤3.5	Continue therapeutic anticoagulation, perform dental procedure at trough concentrations
High bleeding risk dental procedures*	Perform dental procedure without interruption	Perform dental procedure without interruption if INR is ≤3.5 24 h before the intervention. Delay if INR >3.5 and adjust VKA dose until INR ≤3.5	Delay (rivaroxaban, edoxaban) or skip (apixaban, dabigatran) one dose on the morning of the dental intervention

*Application of local haemostatic measures and other preventive strategies recommended, e.g. limiting the surgical site or performing the dental intervention in the morning.

SAPT: single antiplatelet therapy; DAPT: dual antiplatelet therapy; ASA: acetylsalicylic acid; VKA: vitamin K antagonist; DOAC: direct oral anticoagulant; INR: international normalized ratio.

## Dental patients receiving oral anticoagulant therapy

### Vitamin K antagonists (VKA)

The management of patients who require dental interventions and receive chronic treatment with VKAs have been extensively investigated [25–27]. There is general agreement that treatment regimens with VKAs should not be altered before dental procedures [25]. The current guidelines of the American College of Chest Physicians (ACCP) on the perioperative management of antithrombotic therapy recommend dental surgery without VKA interruption with the co-administration of a prohaemostatic agent [1]. British guidelines state that oral anticoagulation with VKA should not be discontinued in the majority of patients requiring dental surgery [28]. Most randomized trials and prospective cohort studies assessing periprocedural anticoagulant management in VKA-treated patients undergoing dental procedures showed similar rates of postoperative bleeding after dental surgery in continuously anticoagulated patients, patients whose anticoagulation was reduced or withdrawn, and non-anticoagulated patients [8–11]. Therefore, most authors concluded that the risk of interrupting or reducing VKA therapy outweighed the consequences of potential bleeding complications. Based on the available evidence and extensive clinical experience, the interruption of VKA treatment before dental procedures is not recommended for interventions that are unlikely to cause bleeding, and for low and high bleeding risk procedures if the INR of the patient is ≤3.5 24 h before the planned intervention. If INR ≥3.5, dose adjustment is required, and the procedure should be delayed until the patient’s INR has been reduced to less than 3.5 [23,29,30]. According to current recommendations, this strategy applies for both low and high bleeding risk dental procedures [2].

### Direct oral anticoagulants (DOAC)

Recently, several direct oral anticoagulants (DOACs) have been developed and tested in large clinical trials as well as real-world studies. These include the direct factor Xa inhibitors rivaroxaban, apixaban and edoxaban, and the direct thrombin inhibitor dabigatran. The new agents are now approved for indications including the acute treatment of deep vein thrombosis (DVT) and pulmonary embolism (PE), the prevention of stroke and systemic embolization in non-valvular atrial fibrillation (NVAF), venous thromboembolism (VTE) prophylaxis after orthopaedic surgery and in hospitalized medically ill patients, and for the management of ACS. For each agent, lower doses are indicated for patients with various levels of renal impairment, and in some cases, for the elderly [31–33].

Due to the lack of standard monitoring to assess the bleeding risk in patients taking DOACs, dental practitioners usually find the management of these patients challenging [34]. Currently, no specific evidence-based guideline recommendations are available for the management of dental patients receiving DOACs [12]. A recent evidence summary has revealed that the research pertaining on dental surgery in patients taking DOACs is of very low quality and limited volume [35].

Practical recommendations and the summary of product characteristics (SmPC) of DOACs contain recommendations for the management of dental patients [2,31–33,36]. The most recent information suggests that simple surgical interventions with a low bleeding risk such as dental extractions do not require the interruption of DOACs in patients with normal renal function (Table 2). Where possible, it is recommended that the procedure is performed at trough concentrations of DOACs, i.e. 12 or 24 h after the last intake, depending on twice-daily or once-daily dosing. Interventions at peak plasma concentration should be avoided [2,32,36]. For patients taking DOACs who require a dental procedure with a higher risk of bleeding complications (Table 1, high bleeding risk procedures), it is recommended to delay the morning dose of once-daily agents (rivaroxaban, edoxaban) on the day of dental treatment, and skip one dose of twice-daily medications (apixaban, dabigatran) [2]. For patients usually taking their rivaroxaban or edoxaban dose in the evening, there is no need to modify their medication schedule before dental treatment [2]. If complete haemostasis has been achieved, DOACs can be resumed six-to-eight hours after the intervention. Due to the short time to peak plasma concentration of DOACs, resuming the drug at the same dose once haemostasis has been established provides a rapid restoration of anticoagulation after the intervention. Therefore, bridging with other anticoagulants is not necessary for patients undergoing dental interventions [37]. In emergency settings, if the required procedure is associated with a high risk of bleeding, referral to an oral surgeon may be necessary [34].

Further practical recommendations for patients undergoing high bleeding risk dental interventions include scheduling the dental treatment for the morning to allow for monitoring and the management of potential bleeding complications, limiting the surgical site by performing a single extraction or limiting subgingival periodontal scaling to three teeth and assessing bleeding before continuing, and the use of haemostatic measures to achieve haemostasis as soon as possible.

Finally, it has to be noted that while the classification of procedures based on the expected risk of bleeding may guide decisions about the continuation or temporary interruption of antithrombotic therapy, management approaches should always be individualized taking into account the patient’s current medication schedule and chronic conditions that may further influence the risk of bleeding (e.g. renal or hepatic impairment, thrombocytopaenia, concomitant anticoagulants, antiplatelets, or non-steroidal anti-inflammatory drugs) as well as the availability of haemostatic measures [34]. Checking for clinically important drug interactions and consultation with a pharmacists or clinical pharmacologist are required during the management of patients taking multiple medications.

## Conclusions

Currently, available evidence suggests that most dental interventions can be safely performed without the interruption of antithrombotic therapy. However, further studies are needed to establish evidence-based guidelines for the periprocedural antithrombotic management of patients receiving direct oral anticoagulants or novel antiplatelet agents.

## Abbreviations

ACCP: American College of Chest Physicians
ACS: acute coronary syndrome
ADP: adenosine diphosphate
BPE: basic periodontal examination
CrCl: creatinine clearance
DAPT: dual antiplatelet therapy
DVT: deep vein thrombosis
DOAC: direct oral anticoagulant
ICD: implantable cardioverter defibrillator
INR: international normalized ratio
NOAC: novel oral anticoagulant
NVAF: non-valvular atrial fibrillation
PCI: percutaneous coronary intervention
PE: pulmonary embolism
RSI: root surface instrumentation
SDCEP: The Scottish Dental Clinical Effectiveness Programme
TURP: transurethral resection of the prostate
VKA: vitamin K antagonist
VTE: venous thromboembolism
